# The intramembrane COOH-terminal domain of PRRT2 regulates voltage-dependent Na^+^ channels

**DOI:** 10.1016/j.jbc.2023.104632

**Published:** 2023-03-22

**Authors:** Francesca Franchi, Antonella Marte, Beatrice Corradi, Bruno Sterlini, Giulio Alberini, Alessandra Romei, Antonio De Fusco, Alexander Vogel, Luca Maragliano, Pietro Baldelli, Anna Corradi, Pierluigi Valente, Fabio Benfenati

**Affiliations:** 1 Center for Synaptic Neuroscience and Technology, Istituto Italiano di Tecnologia, Genova, Italy; 2 Department of Experimental Medicine, University of Genova, Genova, Italy; 3 IRCCS, Ospedale Policlinico San Martino, Genova, Italy; 4 Department of Life and Environmental Sciences, Polytechnic University of Marche, Ancona, Italy

**Keywords:** PRRT2, voltage-dependent sodium channels, molecular dynamics, structure-function relationships, intrinsic excitability

## Abstract

Proline-rich transmembrane protein 2 (*PRRT2*) is the single causative gene for pleiotropic paroxysmal syndromes, including epilepsy, kinesigenic dyskinesia, episodic ataxia, and migraine. PRRT2 is a neuron-specific type-2 membrane protein with a COOH-terminal intramembrane domain and a long proline-rich NH_2_-terminal cytoplasmic region. A large array of experimental data indicates that PRRT2 is a neuron stability gene that negatively controls intrinsic excitability by regulating surface membrane localization and biophysical properties of voltage-dependent Na^+^ channels Nav1.2 and Nav1.6, but not Nav1.1. To further investigate the regulatory role of PRRT2, we studied the structural features of this membrane protein with molecular dynamics simulations, and its structure-function relationships with Nav1.2 channels by biochemical and electrophysiological techniques. We found that the intramembrane COOH-terminal region maintains a stable conformation over time, with the first transmembrane domain forming a helix–loop–helix motif within the bilayer. The unstructured NH_2_-terminal cytoplasmic region bound to the Nav1.2 better than the isolated COOH-terminal intramembrane domain, mimicking full-length PRRT2, while the COOH-terminal intramembrane domain was able to modulate Na^+^ current and channel biophysical properties, still maintaining the striking specificity for Nav1.2 *versus* Nav1.1. channels. The results identify PRRT2 as a dual-domain protein in which the NH_2_-terminal cytoplasmic region acts as a binding antenna for Na^+^ channels, while the COOH-terminal membrane domain regulates channel exposure on the membrane and its biophysical properties.

Proline-rich transmembrane protein 2 (*PRRT2*) is the causative gene for a clinical-genetic spectrum of heterogeneous paroxysmal neurological disorders (1, 2). *PRRT2* mutations account for a large fraction of cases of familial benign infantile epilepsy, paroxysmal kinesigenic dyskinesia (PKD) and PKD with infantile convulsions (PKD/IC) and, conversely, 95% of PRRT2 patients have a diagnosis within the benign familial infantile epilepsy PKD/IC-PKD spectrum. In addition, 5% of PRRT2 patients display other disorders, such as episodic ataxia, hemiplegic migraine, developmental delay, and intellectual disability (3, 4, 5, 6, 7, 8). To date, about 1500 patients with 70 different mutations have been reported, 75% of whom carrying the same frameshift mutation (c.649dupC). About 75% of all reported *PRRT2* mutations involve insertion of a precocious stop codon, leading to unstable mRNA and/or a truncated protein that is degraded, while some missense mutations may lead to PRRT2 mislocalization (9, 10, 11, 12). Patients bearing homozygous or compound heterozygous mutations in *PRRT2* show a severe encephalopathic phenotype, with paroxysmal dyskinesias, unusually prolonged ataxia attacks, seizures, and intellectual disability (13, 14, 15).

A large body of experimental data have contributed to the understanding of PRRT2 function and pathogenesis of paroxysmal disorders associated with loss-of-function mutations in *PRRT2*, that has been described as a network stability gene (16, 17). We and other groups have found that genetic deletion of the *PRRT2* gene in the mouse (PRRT2KO) mimics the human pathology and displays network hyperexcitability particularly in brain areas where PRRT2 is highly expressed, such as the cerebellum and the hippocampus (18, 19, 20, 21, 22, 23, 24, 25). Physiological levels of PRRT2 seem to be necessary to maintain a normal level of network activity, so that an overexpression of PRRT2, occurring in the 16p11.2 duplication, is also associated with neuropsychiatric disorders including epilepsy (26). PRRT2 is composed of a large proline-rich intracellular NH_2_-terminal domain and two hydrophobic segments of which the second one spans the plasma membrane, whereas the first one forms a helix-loop-helix structure within the inner leaflet of the membrane without crossing it completely (27). PRRT2 is enriched in the axon and nerve terminals and its silencing impairs synchronous release by decreasing the Ca^2+^ sensitivity of neurotransmitter release and thereby greatly increasing synaptic facilitation (21, 28, 29, 30).

In addition to the synaptic dysfunction, a major contribution to hyperexcitability is due to the increased Na^+^ current density observed in PRRT2KO mice and induced pluripotent stem cell-derived human neurons from homozygous patients (21, 31). Interestingly, PRRT2 is a physiological inhibitor of Nav1.2/Nav1.6 which predominantly sustain the firing activity of excitatory neurons, while not affecting Nav1.1 channels that play a central role in firing of inhibitory neurons (32, 33). This way, the loss-of-function of PRRT2 unleashes the constraint on excitability of excitatory neurons, generating an excitatory/inhibitory unbalance (16, 18, 21, 31). In heterologous systems, PRRT2 acts as a negative modulator of Nav1.2/1.6 channels by decreasing their targeting to the plasma membrane, shifting the inactivation curve toward more negative voltages and impairing channel recovery after inactivation (31). Many of these effects are opposite to the actions of Nav β-subunits, suggesting that they may result from an antagonistic interaction between PRRT2 and β-subunits. However, no detectable molecular and/or functional interactions were found (34). These findings, supported by the proven efficacy of Na^+^ channel antagonists in PRRT2 patients and mutant mice (35, 36, 37), indicate that PRRT2 directly interacts with the Nav α-subunits.

Here, based on the membrane topology of PRRT2, we studied the conformational features of the bilayer-spanning region with microsecond-time scale molecular dynamics (MD) simulations to validate and further refine our previous structural model (27). These simulations revealed that PRRT2 maintains a stable conformation within the membrane, characterized by the presence of a helix-loop-helix motif, a short cytoplasmic loop, and a transmembrane helix. We then investigated the structure-function relationships of PRRT2 with Nav1.2 channels by biochemical and electrophysiological techniques. We found that both the NH_2_-terminal and COOH-terminal regions of PRRT2 bind Nav1.2, while the latter intramembrane domain is responsible for the modulation the membrane targeting and biophysical properties of Nav1.2 channels. Both PRRT2 regions did not significantly interact with Nav1.1. channels. The results identify PRRT2 as a multidomain protein in which the NH_2_-terminal cytoplasmic region binds the Nav1.2 channel but is unable to modulate it, while the COOH-terminal transmembrane region is responsible for the modulation of the channel fate and properties.

## Results

### PRRT2 deletion mutants are expressed and targeted to the plasma membrane in Hek293 cells

**Figure 1:**
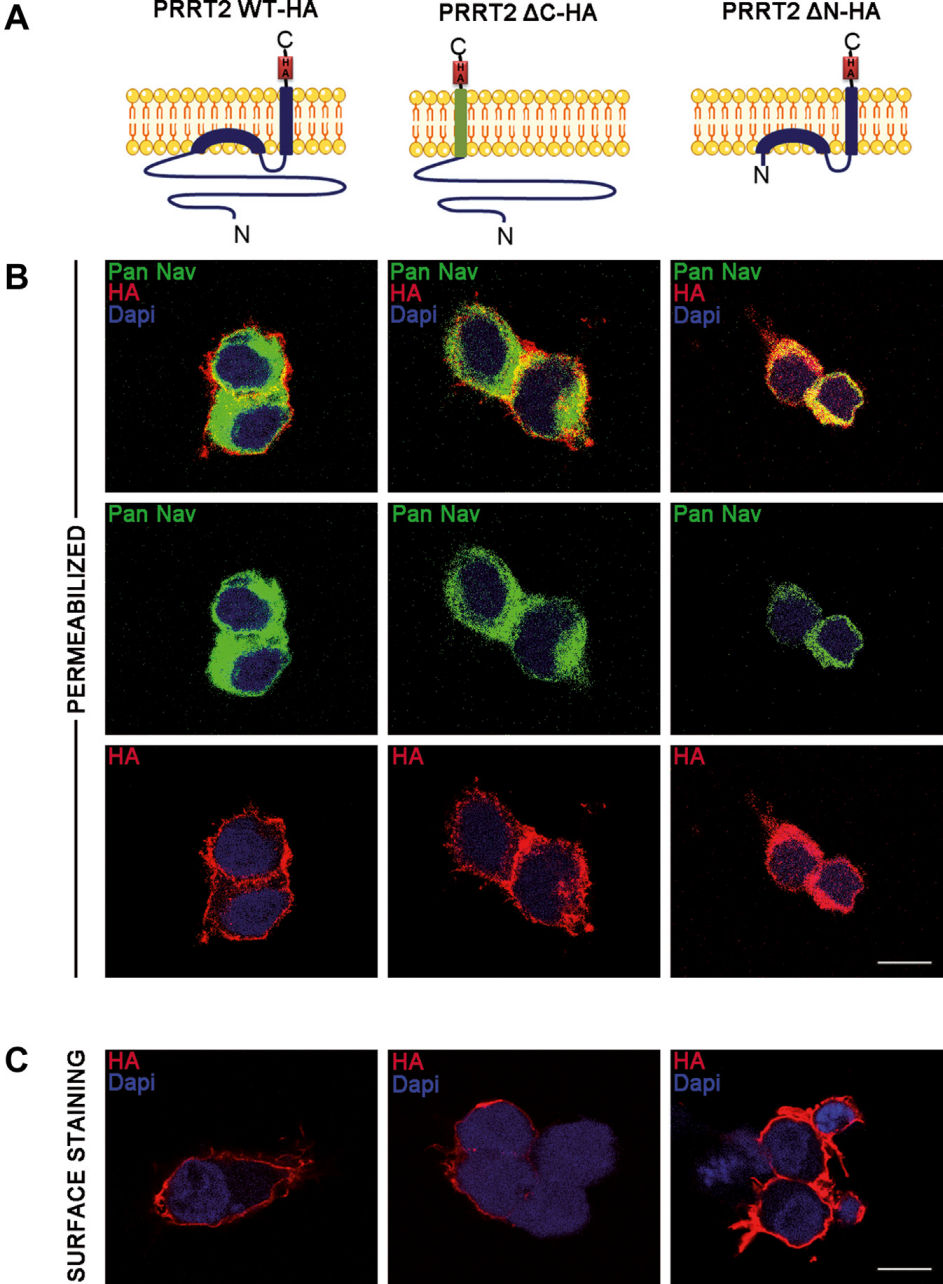
**Generation and characterization of the PRRT2 deletion mutants**. *A*, schematics of the PRRT2 domain constructs. PRRT2 WT-HA is the entire protein (*violet*). PRRT2 ΔC-HA is a chimeric protein composed of the cytoplasmic PRRT2 (*violet*) domain anchored to the membrane by the transmembrane domain of IFITM1 (*green*). PRRT2 ΔN-HA is composed of the transmembrane domain of PRRT2. *B*, Hek- Nav1.2 cells transfected with (from *left to right*) PRRT2-HA, PRRT2ΔC-HA, and PRRT2ΔN-HA were permeabilized and subsequently labeled for anti-HA and pan-Nav antibodies, respectively, with nuclei marked with DAPI. The individual HA and pan-Nav staining are shown together with the respective merge images (*top row*). *C*, Hek-Nav cells transfected with PRRT2-HA, PRRT2ΔC-HA, and PRRT2ΔN-HA were surface-labeled with anti-HA to stain the membrane-exposed domains of the proteins. Scale bar, 10 μm. IFITM1, interferon-induced transmembrane protein 1; *PRRT2*, proline-rich transmembrane protein 2.

Given the specific interaction with Nav1.2/1.6, but not with Nav1.1, channels, it was of interest to investigate whether the transmembrane COOH-terminal region or the cytosolic NH_2_-terminal region of the molecule was responsible for the specific interaction. To this aim, we generated two PRRT2 deletion mutants alternatively lacking the two regions and fused to a HA reporter sequence, namely: PRRT2ΔC-HA, a chimeric protein composed of the NH_2_-terminal cytoplasmic PRRT2 domain anchored to the membrane by the transmembrane domain of the structurally homologous protein interferon-induced transmembrane protein 1 (IFITM1; (38)) and PRRT2ΔN-HA composed of the COOH-terminal transmembrane domain of PRRT2 including the short cytoplasmic loop (Fig. 1*A*). Full-length PRRT2-HA or either mutant construct was then transfected in naïve and Hek293 clones expressing Nav1.2 channels (Figs. 1*B* and S1). All mutants were correctly expressed and did not interfere with Nav1.2 expression (Fig. S1). Moreover, the HA immunoreactivity of all three constructs was found in association with Nav1.2 channels stained with pan-Nav antibodies (Fig. 1*B*). Live staining of nonpermeabilized Hek-Nav1.2 cells with anti-HA antibodies confirmed the membrane targeting of the PRRT2 variants, as deduced from the staining of the surface-exposed COOH-terminal HA (Fig. 1*C*).

### Refinement of the structure of the PRRT2 COOH-terminal region by MD simulations

**Figure 2:**
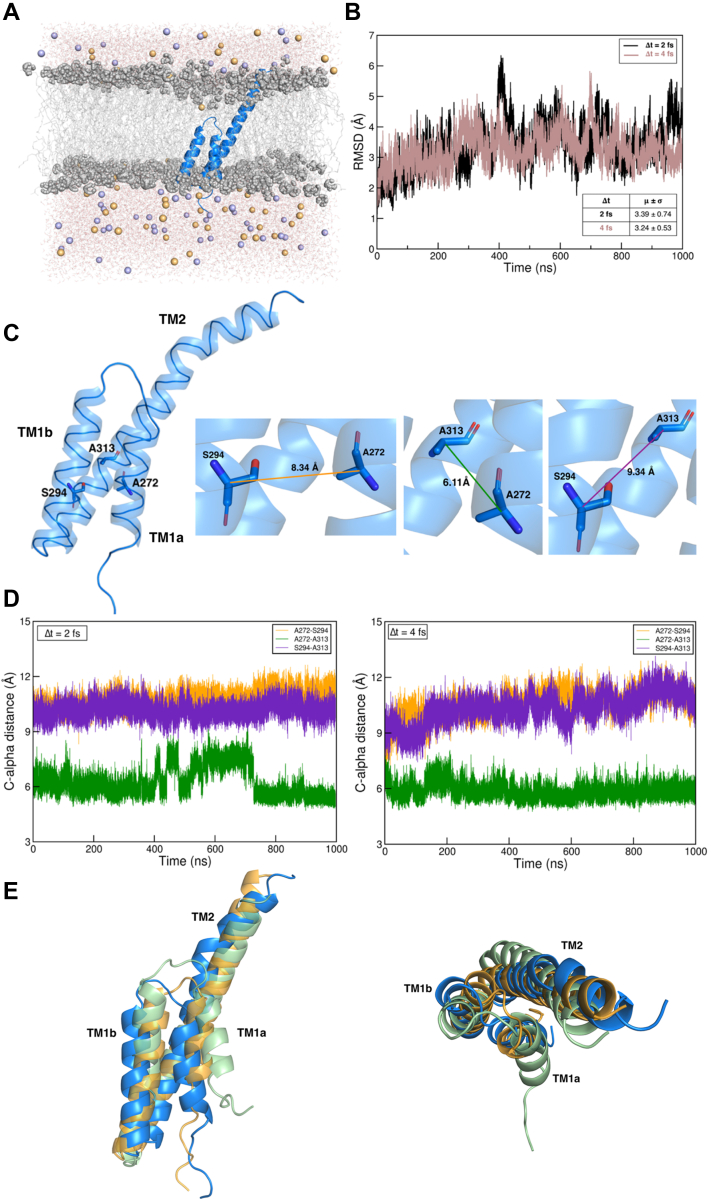
**Molecular modeling ofthePRRT2 transmembrane segment.***A*, snapshot of the simulated system extracted from a trajectory; PRRT2 is represented in *blue cartoon*, phospholipids heads and tails as *gray spheres and sticks*, respectively, water molecules as *red* and *white sticks*, and sodium and chloride ions as *orange and purple spheres*, respectively. *B*, alpha-carbon RMSD of PRRT2 calculated along the two simulated trajectories at different timesteps (Δt); average values and S.D. are indicated. *C*, cartoon representation of the PRRT2 transmembrane domain; residues involved in the calculated cross-distances are represented as *sticks*; the values reported are those at the beginning of the simulations. *D*, cross-distances evolution for PRRT2 trajectories. *E*, lateral and extracellular views of Robetta (*blue*) AlphaFold2 (*green*), and ESMFold (*orange*) PRRT2 models, superimposed *via* PyMOL. *PRRT2*, proline-rich transmembrane protein 2.

We previously reported (27) that PRRT2 is an atypical dyspanin, presenting a long and unstructured intracellular NH_2_-terminal region (residues M1 to R266), a first intramembrane domain (TM1a-b, residues D267 to Q300, intervals according to the OPM server https://opm.phar.umich.edu/) that bends with a short hinge including two proline residues (P279 and P282) to form a helix-loop-helix structure; a full membrane-spanning domain (TM2, residues V303 to G337), and a minimal extracellular COOH-terminal, V338 to K340 (Fig. 2*A*). In our structural model, the C terminus of TM1b (S294 to Q300) and the N terminus of TM2 (V303 to R311) are in the cytosol and, opposite to what obtained in a previous bioinformatic analysis (1), they are connected by only two residues (G301 and D302).

Here, we refined our transmembrane domain model by performing two distinct, 1 μs-long, MD simulations, one using the standard approach with 2 fs timestep, and one with hydrogen mass repartitioning and 4 fs timestep. These simulations demonstrated a high degree of structural stability for the transmembrane PRRT2 segment: the α-carbon PRRT2 root-mean-square deviation from the starting structure was stationary in both replicas after 250 ns (Fig. 2*B*), and the evolution of the cross-distances between A272, S294, and A313, belonging to the three distinct intramembrane stretches, remained stable over time (Figs. 2, *C* and *D* and S2). These results support the previously proposed model of PRRT2, which was based on much shorter simulation times (50 ns; 27). We also compared our transmembrane model, originally obtained by Robetta, with two additional ones predicted by the AlphaFold2 (39) and ESMFold (40) algorithms and found strong structural similarities between them (Fig. 2*E*). Indeed, the AlphaFold2 and ESMFold models have the same characteristic sequence of secondary structure elements as Robetta’s, with almost any differences in their span and relative position between the three conformations. The root-mean-square deviations of the AlphaFold2 and ESMFold models from the Robetta model are 6.62 and 3.46 Å, respectively, values in line with those observed along the simulated trajectory of the latter.

### Both the NH_2_- and COOH-terminal regions of PRRT2 bind Nav1.2 channels

**Figure 3:**
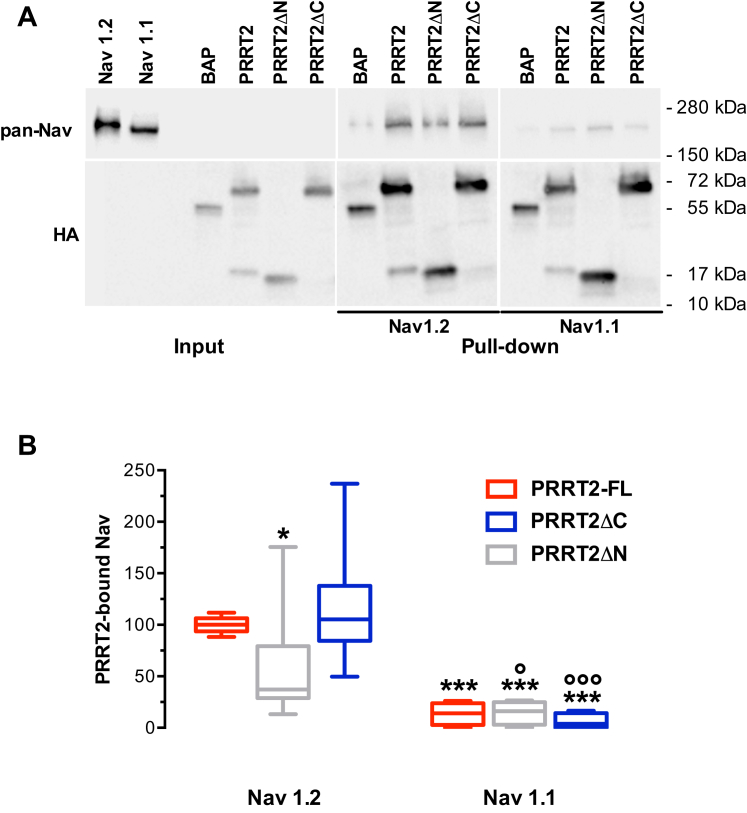
**Binding of PRRT2 deletion mutants to Nav1.2 and Nav1.1 channels**. *A*, representative immunoblot of co-immunoprecipitation of PRRT2 variants. HA-tagged bacterial alkaline phosphatase (BAP), HA-tagged full-length PRRT2 (PRRT2-FL), or its deletion mutants PRRT2ΔN and PRRT2ΔC were expressed in naive Hek293 cells and purified by HA-immunoprecipitation. The extract of Hek293 stable clones expressing either human Nav1.2 or human Nav1.1 was added to the HA-immunoprecipitated BAP, PRRT2, or PRRT2 deletion mutants. Cell lysates (INPUT, 10 μg protein) and samples immunoprecipitated by anti-HA beads were analyzed by Western blotting with pan-Nav and HA antibodies. Molecular mass standards are reported on the right. The representative blots were cut from the same gel. *B*, quantification of the immunoreactive signals in PRRT2-HA immunoprecipitates. Box plots of n = 6 and 4 independent experiments for Hek-Nav1.2 and Hek-Nav1.1, respectively. ∗*p* < 0.05, ∗∗∗*p* < 0.001 *versus* full-length PRRT2; °*p* < 0.05, °°°*p* < 0.001 Nav1.1 *versus* Nav1.2 for each PRRT2 variant. Two-way ANOVA/Fisher’s tests. *PRRT2*, proline-rich transmembrane protein 2.

To ascertain which region of PRRT2 directly interacts with the Nav1.2 α-subunit and the basis for its specificity, we performed affinity binding assays by challenging HA-tagged PRRT2 variants purified from naïve Hek293 cells with extracts of Hek293 clones stably expressing either Nav1.2 or Nav1.1 α-subunits in the absence of β-subunits. After incubation, PRRT2-HA variants were pulled down with anti-HA beads and the associated Nav channels identified by Western blotting with anti-pan-Nav antibodies (Fig. 3*A*). Full-length PRRT2 efficiently pulled down Nav1.2, but not Nav1.1, α-subunits, as previously described (31). The Nav1.2 binding activity was retained when either PRRT2ΔN (COOH-terminal PRRT2) or PRRT2ΔC (NH_2_-terminal PRRT2) were assayed but to a different extent. In fact, while PRRT2ΔC bound Nav1.2 to the same extent of full-length PRRT2, PRRT2ΔN exhibited only about 50% of full-length PRRT2 binding. However, both deletion mutants strictly preserved their binding specificity for Nav1.2 α-subunits, and their binding to the Nav1.1 α-subunit was negligible (Fig. 3*B*).

### The PRRT2 COOH-terminal region decreases the Na^+^ current density in Na_V_1.2-expressing cells

**Figure 4:**
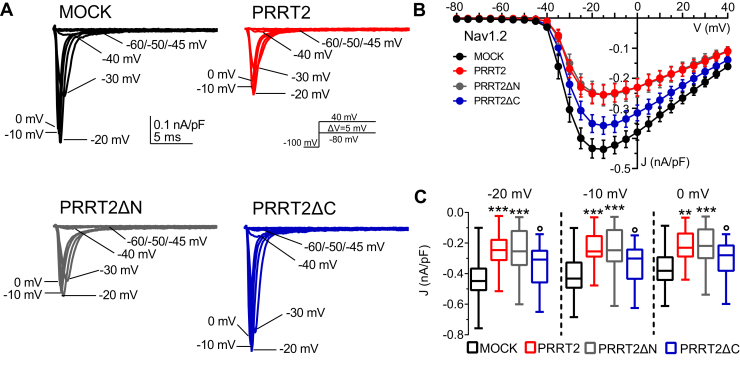
**Effects of PRRT2 deletion mutants on the transient Nav1.2 current**. *A*, representative whole-cell Na^+^ currents recorded in Hek293 cells stably expressing Nav1.2 α-subunits and transiently transfected with empty vector (MOCK, *black*), full-length PRRT2 (*red*), PRRT2ΔN (*gray*), and PRRT2ΔC (*blue*). Currents were elicited by a protocol (*inset*) consisting of 5-mV depolarization steps from −80 to 40 mV from a holding potential of −100 mV. For clarity, the first 20 ms of the 100-ms steps for eight representative traces per condition are plotted. *B*, current density (J) versus voltage (V) relationship for the four experimental conditions. *C*, box plots of J values at three representative voltages (−20/-10/0 mV). MOCK, n = 24; full-length PRRT2, n = 15; PRRT2ΔN, n = 19; PRRT2ΔC, n = 21. ∗∗*p* < 0.01, ∗∗∗*p* < 0.001 *versus* MOCK; °*p* < 0.05 *versus* full-length PRRT2. One-way ANOVA/uncorrected Fisher's LSD test. *PRRT2*, proline-rich transmembrane protein 2.

We next investigated the ability of the PRRT2 deletion mutant to mimic the effects of full-length PRRT2 in inhibiting the transient Na^+^ current in Nav1.2-expressing Hek293 cells analyzed by whole-cell patch-clamp recordings (Fig. 4*A*). HA-tagged PRRT2 variants were expressed to the same extent in Hek-Nav1.2 cells (Fig. S3). As previously reported (31, 34), full-length PRRT2 significantly decreased the Na^+^ current density with respect to cells transfected with the empty vector (MOCK). Interestingly, the effects of PRRT2ΔN were indistinguishable from those of full-length PRRT2, while PRRT2ΔC was substantially ineffective (Fig. 4*B*). The modulation of the Na^+^ current density by full-length PRRT2 and PRRT2ΔN were highly significant in the −20 to 0 mV range (Fig. 4*C*). However, no significant voltage shift of the current density/voltage (J/V) curves was observed (Fig. 4*B*).

### The PRRT2 COOH-terminal region modulates the biophysical properties of Nav1.2 channels

**Figure 5:**
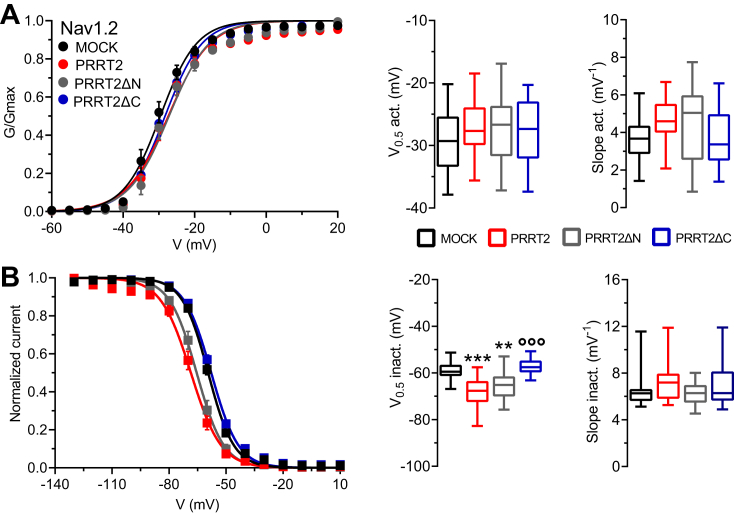
**Effects of PRRT2 deletion mutants on the activation and inactivation kinetics of Nav1.2 channels**. Hek293 cells stably expressing Nav1.2 α-subunits were transiently transfected with empty vector (MOCK, *black*), full-length PRRT2 (*red*), PRRT2ΔN (*gray*), and PRRT2ΔC (*blue*). *A*, *Left:* Voltage-dependence of activation. The lines are the best-fitted Boltzmann curves. *Right:* Box plots of the half-maximal voltage of activation (V_0.5_) and slope (MOCK, n = 24; full-length PRRT2, n = 15; PRRT2ΔN, n = 19; PRRT2ΔC, n = 21). *B*, *Left:* Steady-state inactivation curves. The lines are the best-fitted Boltzmann curves. *Right:* Means (±SEM) values of the half-maximal voltages for inactivation (V_0.5_ inact.) and slopes (MOCK, n = 20; full-length PRRT2, n = 18; PRRT2ΔN, n = 15; PRRT2ΔC, n = 21). ∗∗*p* < 0.01, ∗∗∗*p* < 0.001 *versus* MOCK; °°°*p* < 0.001 *versus* full-length PRRT2. One-way ANOVA/Dunnett’s test. *PRRT2*, proline-rich transmembrane protein 2.

**Figure 6:**
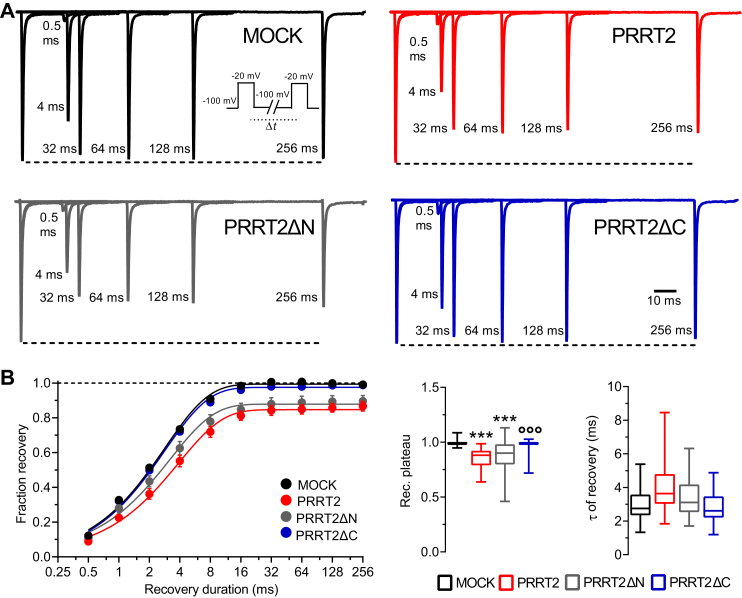
**Effects of PRRT2 deletion mutants on the recovery from inactivation of Nav1.2 channels**. *A*, representative traces showing current recovery from inactivation for all the experimental conditions. Recordings were obtained prepulsing cells to −20 mV for 20 ms to inactivate Na^+^ currents and then coming back to a recovery potential of −100 mV for increasing durations before the repetition of test pulse to −20 mV. For clarity, 6 of the 9 time-intervals are shown. *B*, *left:* the time courses of the recovery from inactivation of peak currents at −20 mV are plotted on a semi-logarithmic scale for the four experimental conditions. *Right:* Box plots of τ and plateau of recovery estimated from one-phase decay fit to the data (MOCK, n = 24; full-length PRRT2, n =16; PRRT2ΔN, n = 18; PRRT2ΔC, n = 21). ∗∗∗*p* < 0.001 *versus* MOCK; °°°*p* < 0.001 *versus* full-length PRRT2. Kruskal–Wallis/Dunn’s tests. *PRRT2*, proline-rich transmembrane protein 2.

In addition to a constraint on the transient Na^+^ current, the expression of PRRT2 also negatively modulated intrinsic excitability by affecting the inactivation and recovery from inactivation of Nav1.2/1.6 channels (31). Here, we investigated the activity of the NH_2_- and COOH-terminal regions of PRRT2 by challenging its deletion mutants with Hek293 cells stably expressing Nav1.2. The activation dynamics of the Nav1.2 α-subunit, that is heavily modulated by the β-subunits (41), is not significantly affected by either the expression of full-length PRRT2 or its deletion mutants with respect to MOCK-transfected cells in terms of voltage sensitivity (slope) and voltage of half-activation (V_0.5_; Fig. 5*A*). On the contrary, full-length PRRT2 induced a significant left shift of the steady-state inactivation curve of Nav1.2 by significantly decreasing the voltage of half-inactivation toward more negative values with respect to MOCK-transfected cells (Fig. 5*B*). The same effect, with similar magnitude, was triggered by the expression of the PRRT2ΔN mutant, while the expression of the PRRT2ΔC mutant was totally ineffective (Fig. 5*B*). Similar effects were observed on the Nav1.2 recovery from inactivation (Fig. 6*A*). The COOH-terminal region of PRRT2 fully recapitulated the significant decrease in the plateau of recovery induced by full-length PRRT2, while the cytosolic NH_2_-terminal region of PRRT2 was ineffective (Fig. 5*B*).

### Both the NH_2_- and COOH-terminal regions of PRRT2 do not interact with Nav1.1 channels

**Figure 7:**
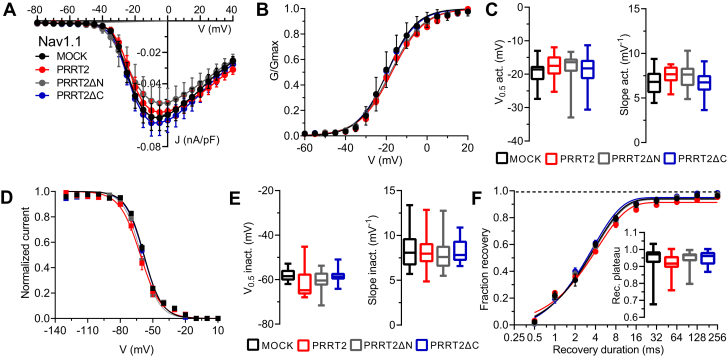
**PRRT2 deletion mutants are ineffective on the transient current and biophysical properties of Nav1.1 channels**. Hek293 cells stably expressing Nav1.1 α-subunits were transiently transfected with empty vector (MOCK, *black*), full-length PRRT2 (*red*), PRRT2ΔN (*gray*), and PRRT2ΔC (*blue*). *A*, current density (J) *versus* voltage (V) relationship for the four experimental conditions, *B*, voltage-dependence of activation. The lines are the best-fitted Boltzmann curves. *C*, box plots of the half-maximal voltage of activation (V_0.5_) and slope (MOCK, n = 21; full-length PRRT2, n = 19; PRRT2ΔN, n = 18; PRRT2ΔC, n = 23). *D*, steady-state inactivation curves. The lines are the best-fitted Boltzmann curves. *E*, box plots of the half-maximal voltages for inactivation (V_0.5_ inact.) and slopes (MOCK, n = 18; full-length PRRT2, n = 18; PRRT2ΔN, n = 16; PRRT2ΔC, n = 18). *F*, the time courses of the recovery from inactivation of peak currents at −20 mV are plotted on a semi-logarithmic scale for the four experimental conditions. *Inset:* Box plots of the plateau of recovery estimated from one-phase decay fit to the data (MOCK, n = 19; full-length PRRT2, n = 18; PRRT2ΔN, n = 17; PRRT2ΔC, n = 18). *p* > 0.05 *versus* MOCK. One-way ANOVA/Dunnett’s test. *PRRT2*, proline-rich transmembrane protein 2.

We next investigated whether the functional specificity of full-length PRRT2 for Nav subtypes was preserved to some extent in its deletion mutants, as suggested by co-immunoprecipitation experiments. Both full-length PRRT2 and well as its PRRT2ΔN and PRRT2ΔC mutants did not significantly affect the density of the transient Na^+^ currents nor its voltage dependence with respect to MOCK-transfected Hek293 cells stably expressing Nav1.1 channels (Fig. 7*A*). Similarly, both full-length PRRT2 and its deletion mutants were totally ineffective in altering the parameters of activation (Fig. 7*B* and *C*), steady-state inactivation (Fig. 7, *D* and *E*), and recovery from inactivation (Fig. 7*F*) curves of Nav1.1 channels.

## Discussion

Given the common paroxysmal character of the pleiotropic diseases linked to PRRT2 loss-of-function (3, 6, 7, 8), the lack of negative modulation of Na^+^ channels can be proposed as the key pathogenetic mechanism, in addition to the excitatory/inhibitory imbalance in short-term plasticity (21, 31). The interactions of PRRT2 with Na^+^ channels not only provide a basis for the pathogenesis of the PRRT2-linked paroxysmal manifestations, but also indicate that PRRT2 could be a "*chameleon-like*", multifunctional protein controlling network stability (16, 17). A strong support to the pathogenic role of the disruption of PRRT2/Nav interactions is provided by the efficacy of Na^+^ channel blockers in the therapy of PRRT2-linked diseases (35, 36, 37). Thus, the PRRT2/Nav interaction is worth investigating to clarify the pathogenesis of the diseases and the genotype-phenotype relationships, as well as to develop new targeted therapies.

A fundamental topic to clarify is the PRRT2 structure-function relationships, *i.e.*, which are the protein domains responsible for the interaction with Nav1.2/1.6 and for affecting their turnover between the plasma membrane and the intracellular stores and their biophysical properties. PRRT2 displays two distinct regions, an NH_2_-terminal unstructured proline-rich region and an intramembrane COOH-terminal region forming a helix-loop-helix a very short cytosolic loop and a transmembrane segment ending with a COOH-terminal tripeptide ((27); this paper).

In the absence of an experimentally determined structure for PRRT2, we previously proposed an atom-detailed model of its 79 amino acid-long intramembrane region, generated using Robetta and refined with short MD simulations (27). Here, to further validate the model, we extended the simulations to the μs-time scale, revealing that the protein maintained a stable conformation with all the distinctive features of the model. We also compared our Robetta model with the structures predicted by the AlphaFold2 and ESMFold programs (39, 40). The three configurations are very similar, showing the same succession of secondary structure patterns with almost identical numbers of helix turns. These results strengthen the validity of the model, which is presently the only structure available for the transmembrane PRRT2 region and is essential to investigate protein–protein interactions in atomic detail.

To define whether a single or both regions of PRRT2 are involved in interacting with Nav and modulating their biophysical properties, we generated NH_2_- and COOH-terminal domain constructs and challenged them with Nav1.2 and Nav1.1 α-subunits to assess the extent and specificity of the interactions. PRRT2 fragments retained the subunit specificity of full-length PRRT2 and did not interact with Nav1.1 channels. Both PRRT2 deletion mutants bound to Nav1.2 α-subunits, although the NH_2_-terminal region displayed a binding comparable to the full-length protein, suggesting an interaction with the cytoplasmic tails and/or intracellular loops of the Nav channel. However, in spite of the recapitulation of the binding activity and Nav channel specificity, the PRRT2 NH_2_-terminal domain was unable to modulate the presence of active channels on the membrane and their biophysical properties.

On the contrary, the COOH-terminal region of PRRT2, whose stable intramembrane 3D structure was defined by MD simulations up to 1 μs simulation time (this paper) and fully confirmed by the recently proposed AlphaFold2 and ESMFold algorithms (39, 40), binds to the channel about half of the full-length form, still preserving the selectivity for the Nav1.2 over the Nav1.1 α-subunit. The persistence of the binding indicates that in addition to cytosolic interactions, the intramembrane domain is another important site of PRRT2/Nav1.2 interaction. The functional importance of this interaction site is testified by the observation that the COOH-terminal region of PRRT2 could entirely recapitulate the effects of the full-length form both on current density (a functional measure of the membrane targeting), inactivation kinetics, and recovery from inactivation of the Nav1.2 channel. These observations restrict the functional effects to a PRRT2/Nav interplay within the plane of the membrane. Notwithstanding the very high homology between the transmembrane domains of Nav1.2 and Nav1.1 α-subunits, the COOH-terminal region of PRRT2 docks to the former and does not interact with the latter. This will open the way to further structural *in silico*, biochemical and electrophysiological investigations by performing site-directed mutagenesis of the nonconserved residues present in the voltage sensing and pore forming regions of the I-IV domains of Nav1.2, using the recently published optimized versions of Nav plasmids (42).

**Figure 8:**
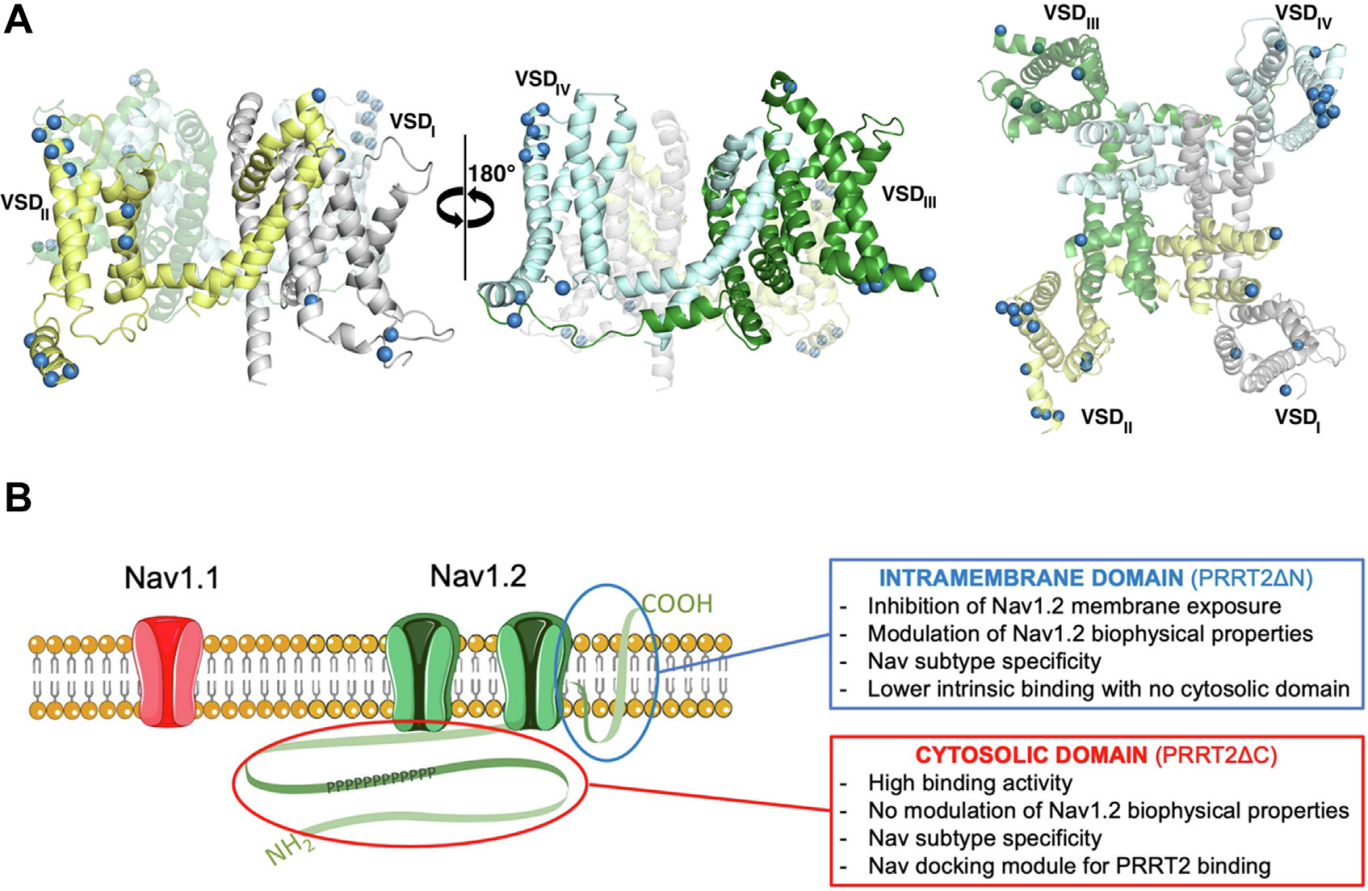
**The tandem domain hypothesis of PRRT2-Nav1.2 specific interactions**. *A*, structure of the transmembrane domains of the human Nav1.2 channel (PDB code: 6J8E; (64)), lateral (*left*) and extracellular (*right*) view. Residues that differ with Nav1.1 are represented as *blue spheres*. *B*, schematic representation of the interactions between Nav1.2 and PRRT2. The NH_2_-terminal intracellular domain may act as a Nav docking module favoring the action of the intramembrane COOH-terminal domain on the plasma membrane exposure and biophysical properties that are specific for Nav1.2. *PRRT2*, proline-rich transmembrane protein 2.

What is then the functional significance of the high intrinsic binding affinity of the cytosolic NH_2_-terminal region of PRRT2? This region is rich in proline residues and displays an intrinsically disordered structure when analyzed by AlphaFold2 (1, 27, 39). However, the significant binding of this PRRT2 region to Nav1.2 *versus* Nav1.1 contributes to the specificity of the interaction. It is possible that, upon binding to the cytosolic domains of Nav1.2, the NH_2_-terminal region of PRRT2 gains a defined structure (43). The marked Nav1.2 binding activity coupled to the inability to modulate current density and biophysical properties of Nav1.2 channels is consistent with a model in which the NH_2_-terminal intracellular domain may act as a Nav1.2 docking module, a PRRT2 “antenna” that favors the action of the intramembrane COOH-terminal domain on the plasma membrane exposure and biophysical properties (Fig. 8). The interaction between the NH_2_-terminal domain of PRRT2 may be direct or mediated by Src-homology-3 domains binding to the proline-rich domain (27) or by the recently reported actin cytoskeleton interactions (44, 45).

In conclusion, the results support the high-order structure of the intramembrane COOH-terminal region of PRRT2 and indicate that intramembrane interactions with the transmembrane domains of Nav1.2 are responsible for the PRRT2 operated constraint of the membrane exposure of intracellular Nav1.2 channels and for decreasing the extent of activation of Nav1.2 channels by shifting the inactivation curve to more negative voltages and decreasing the extent of channel recovery from inactivation. The elucidation of the molecular bases of the inhibitory effects of PRRT2 on neuronal excitability is the first step toward a targeted therapeutic approach to PRRT2-linked paroxysmal diseases aimed at normalizing the intrinsic excitability of principal neurons without a generalized blockade of Nav channels that, although effective, is associated to unwanted side effects.

## Experimental procedures

### Plasmids

The human PRRT2-based constructs used in the study are the following: (i) PRRT2-HA, *i.e.*, pKH3-PRRT2-HA vector encoding for full-length PRRT2 with 3XHA tag fused at the COOH terminus (27); (ii) bacterial alkaline phosphatase (BAP)-HA, *i.e.,* pKH3-BAP-HA vector encoding for BAP with 3XHA tag fused at the COOH terminus (31); (iii) PRRT2ΔN-HA, *i.e.,* pKH3-PRRT2ΔN-HA encoding for the PRRT2 membrane domain (amino acids 261–340) with 3XHA tag fused at COOH terminus (27); (iv) PRRT2ΔC-HA, *i.e*., pKH3-PRRT2ΔN-HA encoding for the PRRT2 cytosolic domain (amino acids 1–260) fused, for membrane targeting, to the second membrane domain of IFTM1(a kind gift of dr. Jacob S. Yount; RRID: Addgene_58415; (45)). For the PRRT2ΔC-HA plasmid, the second membrane domain of IFTM1 was amplified by PCR from pCMV-HA-mIFITM1 with the following primers:

forward, ACGTAAGCTTACCGCCAAGTGCCTGAACAT

reverse, TCAGGTCGACTCTAATGGCACAGACAACGATGAC and cloned in Hind3-Sal1 sites of the pKH3 plasmid (a kind gift of dr. Ian G. Macara, Addgene_12555; (46)). The sequence corresponding to the cytosolic domain of PRRT2 (nucleotides: 1–780) was PCR amplified from pKH3-PRRT2-HA vector with the primers:

forward, CTGAAAGCTTATGGCAGCCAGCAGCTCTGAGATC

reverse, GATCAAGCTTTTCACCCCCCTCCACCCCAGGA and cloned in Hind3 single site of the vector pKH3 encoding the second transmembrane domain of the previously generated IFITM1 (47). The correct orientation and sequence of the cloned fragment were controlled by restriction analysis followed by DNA sequencing.

### Live and conventional cell immunolabeling

Hek-Na_V_1.2 cells transfected with PRRT2-HA, PRRT2ΔC-HA, or PRRT2ΔN-HA constructs were live labeled by diluting primary antibodies (mouse anti-HA, 1:500, Millipore) in culture medium for 30 min at 37 °C/5% CO_2_ to detect surface epitopes, followed by fixation with 4% paraformaldehyde and incubation with Alexa Fluor-594 secondary antibodies. After several washes in phosphate-buffered saline (PBS), coverslips were mounted using Prolong Gold antifade reagent (Invitrogen) containing 4′,6′-diamidino-2-phenylindole for nuclear staining. Hek-Nav1.2 cells transfected with PRRT2-HA, PRRT2ΔC-HA or PRRT2ΔN-HA constructs were fixed in 4% paraformaldehyde at room temperature for 20 min, washed in PBS, and blocked with 10% bovine serum albumin (BSA) for 20 min. Samples were sequentially incubated with mouse anti-pan-Nav (Sigma; 1:100 in 5% BSA) and rabbit anti-HA, (Invitrogen; 1:500 in 5% BSA) primary antibodies, followed by Alexa 564-conjugated or 488-conjugated secondary antibodies (Invitrogen; 1:200 in 5% BSA) at room temperature. After several washes in PBS, coverslips were mounted using Prolong Gold antifade reagent (Invitrogen) containing 4′,6′-diamidino-2-phenylindole for nuclear staining.

### Cell cultures and transfection

Hek293 cells stably expressing human Nav1.2 or Nav1.1 were kind gifts from Drs. Enzo Wanke and Marzia Lecchi (Milano-Bicocca University). To map the PRRT2/Nav interactions, Hek-Nav cells were transiently transfected with the empty pKH3 vector (MOCK), BAP-HA, full-length PRRT2-HA, or its deletion mutants PRRT2ΔN-HA and PRRT2ΔC-HA. All Hek293 cell lines were maintained in DMEM/F12 (1:1) supplemented with 10% fetal bovine serum, 100 U/ml penicillin, 100 μg/ml streptomycin and, for selection of stable Nav clones, 500 μg/ml G418. Cell lines were transfected with 2 μg of each plasmid according to the manufacturer’s recommendations at 70% confluency using Lipofectamine 2000. All reagents were purchased from ThermoFisher Scientific. To identify transfected cells for electrophysiology, the reporter EGFP was co-transfected. Transfected cells were dissociated, re-plated at low density about 24 h posttransfection and recorded after other 24 h.

### Pull-down assays

Naïve Hek293 cells expressing the PRRT2 variants were harvested in lysis buffer (150 mM NaCl, 50 mM Tris, 1 mM EDTA and 1% Triton X-100 supplemented with protease inhibitor cocktail) 48 h after transfection and centrifuged at 10,000*g* for 10 min at 4 °C. Kept an aliquot for input, the supernatant was incubated with 50 μl of monoclonal anti-HA-agarose affinity beads (Sigma-Aldrich) for 2 h at 4 °C. After three washes in lysis buffer, the beads were incubated with cell extracts from Hek293 cells expressing either Nav1.2 or Nav1.1 for 2 h at 4 °C. After extensive washes in lysis buffer and detergent-free lysis buffer, samples were resolved by sodium dodecyl sulfate polyacrylamide gel electrophoresis and subjected to Western blotting with pan-Nav (1:300, Sigma-Aldrich Cat.S8809, RRID:AB_477552) and HA (1:1000, Thermo Fisher Scientific Cat.71–5500, RRID:AB_2533988) specific antibodies. Actin immunoreactivity (anti-actin antibody 1:1000, Sigma-Aldrich Cat.A4700, RRID:AB_476730) was used as control of equal loading.

### Western blotting

For Western blotting analysis, samples were subjected to sodium dodecyl sulfate polyacrylamide gel electrophoresis after 5-min heating at 50 °C and blotted onto nitrocellulose membranes (Whatman). Blotted membranes were blocked for 1 h in 5% milk in Tris-buffered saline (10 mM Tris, 150 mM NaCl, pH 8.0) plus 0.1% Triton X-100 and incubated overnight at 4 °C with the appropriate primary antibody. Membranes were washed and incubated at room temperature for 1 h with peroxidase-conjugated secondary antibodies. The proteins of interest were revealed with the ECL chemiluminescence detection system (Bio-Rad).

### Patch-clamp recordings

Patch pipettes prepared from thin-borosilicate glass (Hilgenberg) were pulled and fire-polished to a final resistance of 2 to 4 MΩ when filled with standard internal solution. Whole-cell currents were recorded using an EPC-10 amplifier (HEKA Electronic). Recordings with leak currents >200 pA or series resistance (R_s_) >10 MΩ were discarded. Data acquisition was performed using PatchMaster program (HEKA Elektronic). R_s_ was compensated 80% (2 μs response time), and the compensation was checked and eventually readjusted before each stimulation protocol. No significant differences in average uncompensated R_s_ errors were found in the cells belonging to the four experimental groups. All recordings were performed at 22 to 24 °C. Voltage-clamp recordings of voltage-gated Na^+^ currents were performed using the following solutions: extracellular (in mM): 140 NaCl, 3 KCl, 1 MgCl_2_, 1 CaCl_2_, 10 Hepes, 10 Mannitol (pH 7.3 with NaOH); intracellular (in mM): 140 CsCl, 10 NaCl, 2 EGTA, 10 Hepes (pH 7.3 with CsOH). Whole-cell family currents of fast inactivating Nav channels were evoked by 5 mV steps depolarization from −80 to 40 mV, and cells were held at −100 mV. Steady-state inactivation curves were constructed by recording the peak currents amplitude evoked by 20-ms test pulses to −10 mV after 500-ms pre-pulses to potentials over the range of −130 to 20 mV. The Na^+^ current density (J) was obtained by dividing the peak inward current by the cell capacitance (nA/pF). The conductance/voltage relationship (G-V) curves were obtained by converting the maximal current values, evoked with the voltage step protocols, to conductance using the relation G_Na_ = I_Na_/(V-E_Na_), where G_Na_ is the Na^+^ conductance, I_Na_ is the peak Na^+^ current, V is the command pulse potential, and E_Na_ is the theoretical reversal potential of Na^+^ current calculated by Nernst equation. G-V curves were normalized and fitted with the Boltzmann function G/G_max_ = 1/(1 + exp[(V − V_1/2_)/k]), where G is the conductance, G_max_ is the maximal conductance, V_1/2_ is the half-maximal voltage of activation, and k is the slope factor. Inactivation curves were fitted with the Boltzmann equation in the following form: 1/[1 + exp(V_1/2_ − V)/k]. Time-dependent rate of recovery from inactivation was calculated by prepulsing the cell with a 20-ms step to −20 mV to inactivate Na^+^ channels and then bringing back the potential to −100 mV for increasing recovery durations (0.5, 1, 2, 4, 8, 32, 64, 128, and 256 ms) before the test pulse of −20 mV. Time constants for recovery from inactivation were calculated by fitting data from each recorded cell to a first order exponential function and averaging time constants across cells. To minimize space-clamp problems, we recoded only isolated transfected cells with a soma diameter of about <30 μm. Membrane capacitance artifacts and leakage currents were eliminated by P/N leak subtraction procedure. For all electrophysiological experiments, data acquisition was performed using PatchMaster programs (HEKA Elektronic).

### Structural models

Our first model structure of the PRRT2 transmembrane domain (sequence G261 to K340) was generated by Rossi *et al*. (27) using the Robetta (48) server, which uses the Rosetta software (49) to predict models of protein domains by combining template-based homology modeling and *de novo* approaches. Two additional models of the same protein segment were generated using AlphaFold2 (39) and ESMFold (40).

### MD simulations

MD simulations were performed using NAMD3.0 (50) with the all-atom CHARMM36m force field (51, 52). The membrane builder application of the CHARMM-GUI server (53, 54) was used for the preparation of all the input files. The Robetta model was oriented with the PPM web server (55), inserted into a 1-palmitoyl-2-oleoyl-sn-glycero-3-phosphocholine lipid bilayer and solvated with water molecules, using the TIP3P model (56). Na^+^ and Cl^-^ ions were added to neutralize the total system charge at a physiological concentration of 0.15 M. The whole system counted 108,608 atoms in a rectangular box. Periodic boundary conditions were applied to replicate the system and remove box surface effects, and the particle mesh Ewald method was used for long-range electrostatics (57), with a grid spacing of 1 Å and sixth-order B-splines. A cut-off of 12 Å and smooth switching at 10 Å was used for Lennard–Jones interactions. Chemical bond distances involving hydrogen atoms were constrained using the SHAKE/RATTLE algorithm (58). Before production, the system was energy minimized, and later, it was equilibrated by running a 15 ns-long simulation in the isobaric-isothermal ensemble, NpT, with N total number of atoms, P = 1 atm and T = 310 K, using positional restraints on the protein atoms. Subsequently, two independent 1 μs-long MD simulations were produced in the NPT ensemble at the same temperature and pressure maintained by a Langevin thermostat and Nosé-Hoover Langevin piston pressure control (59). A timestep of 2 fs was employed for one replica, while the hydrogen mass repartitioning method (HMR) (60) was employed to allow the use of a 4 fs timestep for the second one (61). The MD trajectories were inspected and analyzed to validate the structural stability of the PRRT2 protein using VMD (62) and PyMOL (63). Alpha carbon root mean square deviation and cross-distances analysis were performed for PRRT2 replicas trajectories through tcl scripting (62).

### Statistical analysis

Experimental data are expressed as box plots for the number of independent preparations detailed in the figure legends. The box plots elements are the following: center line, median (Q2); box limits, 25th (Q1)-75th (Q3) percentiles; whiskers, min to max values. Normal distribution of data was assessed using the D’Agostino-Pearson’s normality test. To compare more than two normally distributed sample groups, one-way ANOVA, followed by *post hoc* multiple comparison tests was used. In cases in which data were not normally distributed, nonparametric one-way ANOVA (Kruskal–Wallis’ test) followed by the Dunn’s multiple comparison test was used. Alpha levels for all tests were 0.05% (95% confidence intervals). Statistical analysis was carried out using Prism (GraphPad Software, Inc.) software. In addition to the box plots, data are summarized as means ± SD for the interactions of PRRT2 with Nav1.2 and Nav1.1 in Tables S1 and S2, respectively. The exact *p* values from the statistical analyses are reported in Table S3.

## Data availability

The datasets generated and/or analyzed in the current study are available from the corresponding author on reasonable request.

## Supporting information

This article contains supporting information.

## Conflict of interest

The authors declare that they have no conflicts of interest with the contents of this article.
