# Supplementary material for: The intramembrane COOH-terminal domain of PRRT2 regulates voltage-dependent Na+ channels
Source: J Biol Chem. 2023 Mar 22;299(5):104632. doi: 10.1016/j.jbc.2023.104632 (PMC10164911; doi:10.1016/j.jbc.2023.104632)
Supplement: Supplemental data [file mmc1.pdf]

The intramembrane COOH-terminal domain of PRRT2 regulates voltage-dependent Na^+ channels

Francesca Franchi[1,2], Antonella Marte[2,3], Beatrice Corradi[1,2], Bruno Sterlini[2], Giulio Alberini[1,3], Alessandra Romei[1], Antonio De Fusco[1], Alexander Vogel[1], Luca Maragliano[1,4], Pietro Baldelli[2,3], Anna Corradi[2,3], Pierluigi Valente[2,3], Fabio Benfenati[1,3]

[1] Center for Synaptic Neuroscience and Technology, Istituto Italiano di Tecnologia, Largo Rosanna Benzi 10, 16132 Genova, Italy;
[2] Department of Experimental Medicine, University of Genova, Viale Benedetto XV, 3, 16132 Genova, Italy;
[3] IRCCS, Ospedale Policlinico San Martino, Largo Rosanna Benzi 10, 16132 Genova, Italy;
[4] Department of Life and Environmental Sciences, Polytechnic University of Marche, Ancona, Italy.

SUPPLEMENTARY MATERIALS

SUPPLEMENTARY FIGURES

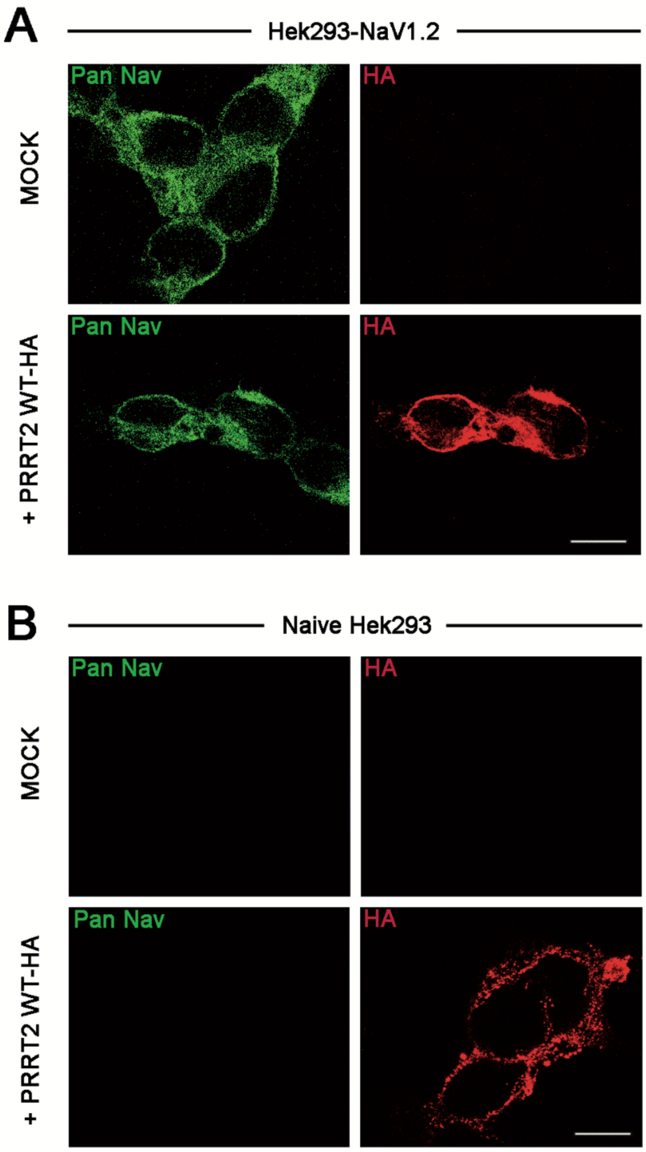

Supplementary Figure 1. Expression of PRRT2-WT in naïve and Nav1.2-expressing Hek293 cells.

Stable Hek293 clones expressing human Nav1.2 channel (**A**) or naïve Hek293 cells (**B**) were transfected with either the empty vector (mock) or a vector encoding full length HA-tagged PRRT2 (PRRT2 WT-HA). Cells were permeabilized and subsequently labeled for anti-HA and pan-Na_V antibodies, respectively. Note the similar distribution of Nav1.2 immunoreactivity in mock and PRRT2 transfected cells, as well as the similar PRRT2 expression pattern in Nav1.2-negative and Nav1.2-expressing cells. Scale bars, 10 μm.

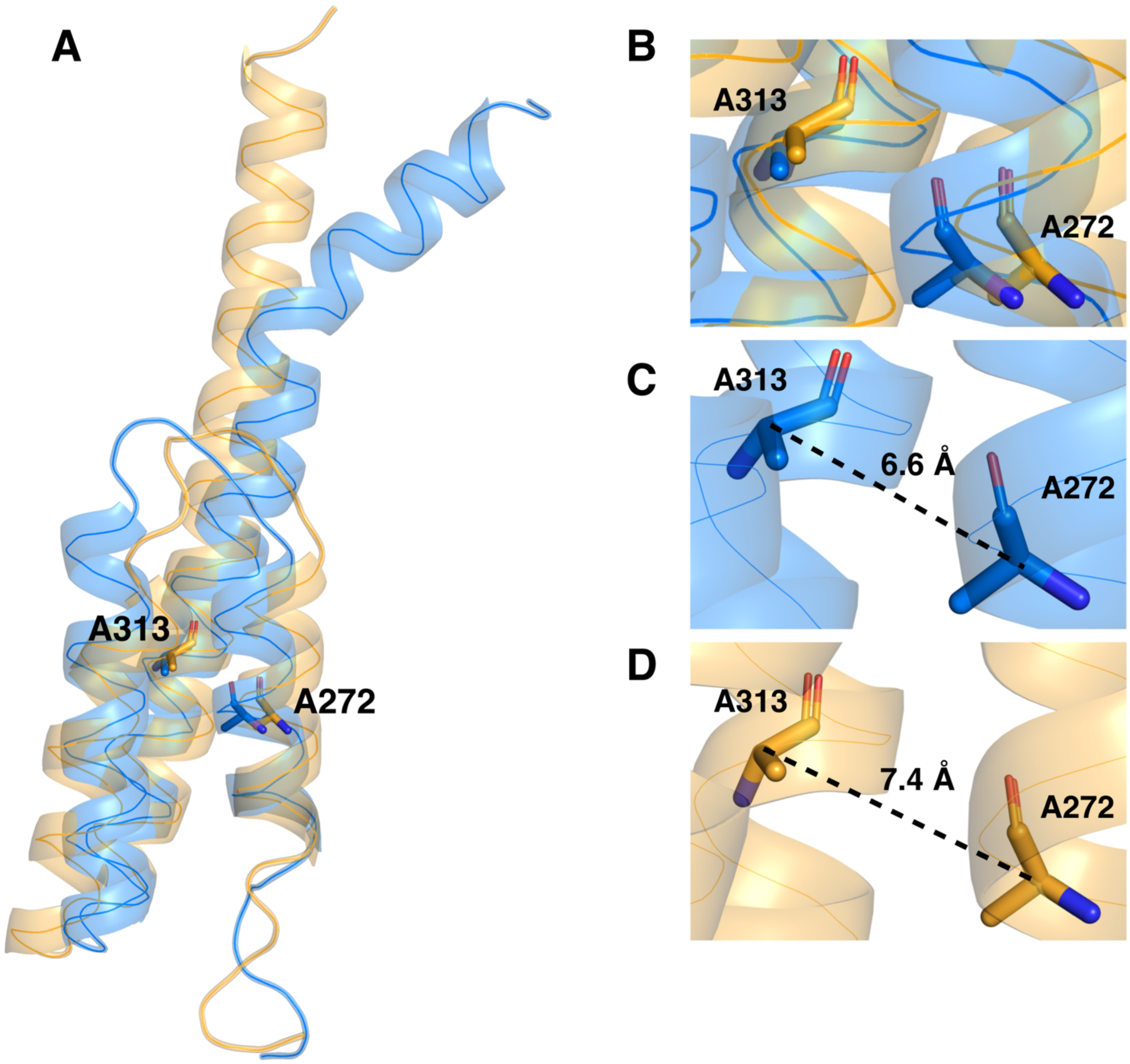

Supplementary Figure 2. A272-A313 Cα distance comparison between PRRT2 configurations at 0 ad 600 ns.

A. Comparison between the PRRT2 transmembrane domain conformation at 600 ns of the 2fs timestep trajectory (orange), corresponding to a larger A272-A313 distance (see Figure 2D in the main text), and the one at the beginning of the simulation (blue). **B.** Enlarged view of A272 and A313 in the superimposed conformations. **C,D.** Distances between the residues in the starting and 600-ns conformation, respectively.

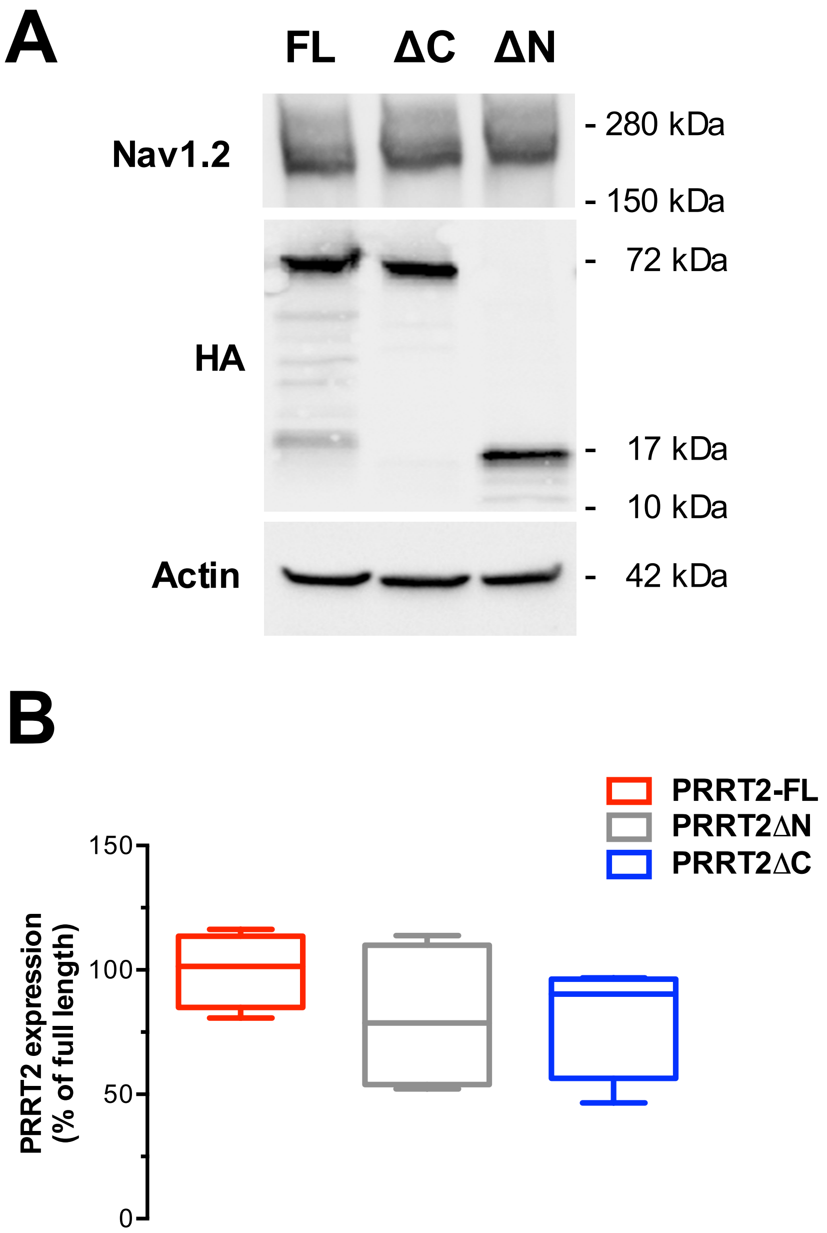

Supplementary Figure 3. Expression of full length PRRT2 and its deletion mutants in Hek293-Nav1.2 cells.

A. HA-tagged full-length PRRT2 (PRRT2-FL) or its deletion mutants PRRT2ΔN and PRRT2ΔC were transfected in Hek293 cells stably expressing Nav1.2 channels. Cell lysates (10 µg protein) were analyzed by western blotting with pan-Na_V and HA antibodies. Actin immunoreactivity was used as a control of equal loading. The representative blots were cut from the same gel. Molecular mass standards are reported on the right. **B.** Quantification of HA immunoreactivity. Box plots of n = 4 independent experiments.

SUPPLEMENTARY TABLES

Supplementary Table 1.
Summary of the results of the PRRT2 interactions with Nav1.2

Parameter	MOCK	PRRT2	PRRT2ΔN	PRRT2ΔC
α-subunit binding (% of PRRT2)	/	100±7.30 (N=6)	58.96±49.50 (N=6) °	116.11±60.39 (N=6) NS
J at -20 mV (nA/pF)	-0.43±0.16 (N=24)	-0.25±0.13 (N=15) ***	-0.24±0.15 (N=19) ***	-0.35±0.14 (N=21) °
J at -10 mV (nA/pF)	-0.42±0.14 (N=24)	-0.25±0.12 (N=15) ***	-0.25±0.15 (N=19) ***	-0.35±0.14 (N=21) °
J at 0 mV (nA/pF)	-0.38±0.13 (N=24)	-0.23±0.11 (N=15) ***	-0.22±0.13 (N=19) ***	-0.32±0.12 (N=21) °
$V_{0.5}$ activation (mV)	-29.41±4.81 (N=24)	-27.27±4.57 (N=15) NS	-27.24±5.47 (N=19) NS	-28.33±5.37 (N=21) NS
Slope activation (mV^{-1})	3.69±1.30 (N=24)	4.584±1.33 (N=15) NS	4.456±2.12 (N=19) NS	3.68±1.39 (N=21) NS
$V_{0.5}$ inactivation (mV)	-59.28±3.41 (N=20)	-68.66±6.33 (N=18) ***	-65.46±5.79 (N=15) **	-57.37±3.49 (N=21) °°°
Slope inactivation (mV^{-1})	6.54±1.45 (N=20) NS	7.22±1.62 (N=18) NS	6.25±0.89 (N=15) NS	6.95±1.93 (N=21) NS
Plateau recovery	0.99±0.03 (N=24)	0.84±0.11 (N=16) ***	0.88±0.14 (N=18) ***	0.97±0.06 (N=21) °°°
Tau recovery (ms)	2.87±0.97 (N=24)	3.97±1.61 (N=16) NS	3.45±1.29 (N=18) NS	2.86±0.90 (N=21) NS

NS, not significant; **p<0.01, ***p<0.001 *vs* MOCK; °p<0.05, °°°p<0.001 *vs* full-length PRRT2. One-way ANOVA/Dunnett’s or Fisher’s tests.

Supplementary Table 2.
Summary of the results of the PRRT2 interactions with Nav1.1

Parameter	MOCK	PRRT2	PRRT2ΔN	PRRT2ΔC
α-subunit binding (% PRRT2-Nav1.2)	/	13.58±10.98 (N=4)	14.70±11.41 (N=4) NS	6.06±7.87 (N=4) NS
$V_{0.5}$ activation (mV)	-19.37±3.68 (N=21)	-17.63±3.66 (N=19) NS	-17.83±4.63 (N=18) NS	-19.21±4.76 (N=23) NS
Slope activation (mV^{-1})	6.77±1.43 (N=21)	7.57±0.99 (N=19) NS	7.46±1.29 (N=18) NS	6.70±1.42 (N=23) NS
$V_{0.5}$ inactivation (mV)	-58.18±2.63 (N=18)	-61.21±7.24 (N=18) NS	-60.51±4.22 (N=16) NS	-58.4±3.38 (N=18) NS
Slope inactivation (mV^{-1})	8.45±2.24 (N=18)	8.27±2.17 (N=18) NS	8.01±1.84 (N=16) NS	8.18±1.31 (N=18) NS
Plateau recovery	0.94±0.08 (N=19)	0.91±0.05 (N=18) NS	0.94±0.06 (N=17) NS	0.95±0.04 (N=18) NS

NS, not significant; **p<0.01, ***p<0.001 *vs* MOCK; °p<0.05, °°°p<0.001 *vs* full-length PRRT2. One-way ANOVA/Dunnett's tests.

Supplementary Table 3. Statistical analysis and exact *p* values

	Parameter	Test	Comparison	P value	F (DFn,DFd)
FIGURE 3	PRRT2-Nav interaction	Two-way ANOVA Fisher's LSD test	Anova table Interaction	P=0.1082	$F_{(2,33)}$ = 2.38
			Anova table Row Factor (Nav subtype)	P=0.0001	$F_{(1,33)}$ = 41.29
			Anova table Column Factor (PRRT2 variants)	P=0.2495	$F_{(2,33)}$ = 1.45
			Nav1.2 PRRT2 vs Nav1.2 ΔN	P=0.0186	
			Nav1.2 PRRT2 vs Nav1.2 ΔC	P=0.3295	
			Nav1.2 PRRT2 vs Nav1.1 PRRT2	P=0.0003	
			Nav1.2 PRRT2 vs Nav1.1 ΔN	P=0.0003	
			Nav1.2 PRRT2 vs Nav1.1 ΔC	P=0.0001	
			Nav1.2 ΔN vs Nav1.1 ΔN	P=0.0492	
			Nav1.2 ΔC vs Nav1.1 ΔC	P<0,0001	
FIGURE 4	J at -20mV	One-way ANOVA Fisher's LSD test	ANOVA table	P=0.0001	$F_{(3,75)}$ = 7.85
			MOCK vs PRRT2	P=0.0003	
			MOCK vs ΔN	P<0.0001	
			MOCK vs ΔC	P=0.0655	
			PRRT2 vs ΔN	P=0.8461	
			PRRT2 vs ΔC	P=0.0474	
			ΔN vs ΔC	P=0.0206	
	J at -10mV	One-way ANOVA Fisher's LSD test	ANOVA table	P=0.0002	$F_{(3,75)}$ = 7.65
			MOCK vs PRRT2	P=0.0003	
			MOCK vs ΔN	P=0.0001	
			MOCK vs ΔC	P=0.1012	
			PRRT2 vs ΔN	P=0.9777	
			PRRT2 vs ΔC	P=0.0292	
			ΔN vs ΔC	P=0.0187	
	J at 0mV	One-way ANOVA Fisher's LSD test	ANOVA table	P=0.0003	$F_{(3,75)}$ = 6.96
			MOCK vs PRRT2	P=0.0006	
			MOCK vs ΔN	P=0.0002	
			MOCK vs ΔC	P=0.1078	

			PRRT2 vs ΔN	P=0.9104	
			PRRT2 vs ΔC	P=0.0452	
			ΔN vs DC	P=0.0244	
FIGURE 5	$V_{0.5}$ activation	One-way ANOVA Dunnett's test	ANOVA table	P=0.4312	$F_{(3,79)} = 0.93$
			MOCK vs PRRT2	P=0.4340	
			MOCK vs ΔN	P=0.3623	
			MOCK vs ΔC	P=0.8192	
			PRRT2 vs ΔN	P>0.9999	
			PRRT2 vs ΔC	P=0.8533	
			ΔN vs ΔC	P=0.8339	
	Slope activation	One-way ANOVA Dunnett's test	ANOVA table	P=0.1487	$F_{(3,75)} = 1.83$
			MOCK vs PRRT2	P=0.2131	
			MOCK vs ΔN	P=0.2756	
			MOCK vs ΔC	P>0.9999	
			PRRT2 vs ΔN	P=0.9893	
			PRRT2 vs ΔC	P=0.2036	
			ΔN vs ΔC	P=0.2758	
	$V_{0.5}$ inactivation	One-way ANOVA Dunnett's test	ANOVA table	P<0.0001	$F_{(3,70)} = 22.69$
			MOCK vs PRRT2	P<0.0001	
			MOCK vs ΔN	P=0.001	
			MOCK vs ΔC	P=0.4483	
			PRRT2 vs ΔN	P=0.1494	
			PRRT2 vs ΔC	P<0.0001	
			ΔN vs ΔC	P<0.0001	
	Slope inactivation	Kruskal-Wallis Dunn's test	ANOVA table	P=0.3099	
			MOCK vs PRRT2	P=0.6007	
			MOCK vs ΔN	P>0.9999	
			MOCK vs ΔC	P>0.9999	
			PRRT2 vs ΔN	P=0.6299	
			PRRT2 vs ΔC	P>0.9999	
			ΔN vs ΔC	P>0.9999	
FIGURE 6	Plateau recovery	Kruskal-Wallis Dunn's test	ANOVA table	P<0.0001	
			MOCK vs PRRT2	P<0.0001	
			MOCK vs ΔN	P=0.0010	
			MOCK vs ΔC	P>0.9999	
			PRRT2 vs ΔN	P>0.9999	
			PRRT2 vs ΔC	P=0.0002	
			ΔN vs ΔC	P=0.0145	

	Tau recovery	Kruskal-Wallis<br>Dunn's test	ANOVA table	P=0.0365	
			MOCK vs PRRT2	P=0.0715	
			MOCK vs ΔN	P>0.9999	
			MOCK vs ΔC	P>0.9999	
			PRRT2 vs ΔN	P>0.9999	
			PRRT2 vs ΔC	P=0.0773	
			ΔN vs ΔC	P>0.9999	
FIGURE 7	$V_{0.5}$ activation	Kruskal-Wallis<br>Dunn's test	ANOVA table	P=0.2166	
			MOCK vs PRRT2	P=0.8147	
			MOCK vs ΔN	P=0.4002	
			MOCK vs ΔC	P>0.9999	
			PRRT2 vs ΔN	P>0.9999	
			PRRT2 vs ΔC	P>0.9999	
			ΔN vs ΔC	P=0.9804	
	Slope activation	One-way ANOVA<br>Dunn's test	ANOVA table	P=0.0716	$F_{(3,77)}$ = 2.43
			MOCK vs PRRT2	P=0.1402	
			MOCK vs ΔN	P=0.2471	
			MOCK vs ΔC	P=0.9966	
			PRRT2 vs ΔN	P=0.9872	
			PRRT2 vs ΔC	P=0.0888	
			ΔN vs ΔC	P=0.1663	
	$V_{0.5}$ inactivation	One-way ANOVA<br>Dunnett's test	ANOVA table	P=0.1508	$F_{(3,66)}$ = 1.83
			MOCK vs PRRT2	P=0.1439	
			MOCK vs ΔN	P=0.3466	
			MOCK vs ΔC	P=0.9979	
			PRRT2 vs ΔN	P=0.9505	
			PRRT2 vs ΔC	P=0.1906	
			ΔN vs ΔC	P=0.4196	
	Slope inactivation	Kruskal-Wallis<br>Dunn's test	ANOVA table	P=0.9284	
			MOCK vs PRRT2	P>0.9999	
			MOCK vs ΔN	P>0.9999	
			MOCK vs ΔC	P>0.9999	
			PRRT2 vs ΔN	P>0.9999	
			PRRT2 vs ΔC	P>0.9999	
			ΔN vs ΔC	P>0.9999	
	Plateau recovery	Kruskal-Wallis<br>Dunn's test	ANOVA table	P=0.109	
			MOCK vs PRRT2	P=0.1735	
			MOCK vs ΔN	P>0.9999	

		MOCK vs ΔC	P>0.9999
		PRRT2 vs ΔN	P=0.6715
		PRRT2 vs ΔC	P=0.2349
		ΔN vs ΔC	P>0.9999
